# Psychometric properties of the adapted instrument European Health Literacy Survey Questionnaire short-short form

**DOI:** 10.1590/1518-8345.4362.3436

**Published:** 2021-07-02

**Authors:** Fábio Luiz Mialhe, Katarinne Lima Moraes, Fernanda Maria Rovai Bado, Virginia Visconde Brasil, Helena Alves De Carvalho Sampaio, Flávio Rebustini

**Affiliations:** 1 Universidade Estadual de Campinas, Faculdade de Odontologia de Piracicaba, Piracicaba, SP, Brazil.; 2 Universidade Federal de Jataí, Curso de Enfermagem, Jataí, GO, Brazil.; 3 Secretaria Municipal de Saúde, Piracicaba, SP, Brazil.; 4 Universidade Federal de Goiás, Faculdade de Enfermagem, Goiânia, GO, Brazil.; 5 Universidade Estadual do Ceará, Benfica, Fortaleza, CE, Brazil.; 6 Universidade de São Paulo, Escola de Artes, Ciências e Humanidades, São Paulo, SP, Brazil.

**Keywords:** Health Literacy, Health Promotion, Validation Study, Surveys and Questionnaires, Unified Health System, Adult, Letramento em Saúde, Promoção da Saúde, Estudo de Validação, Inquéritos e Questionários, Sistema Único de Saúde, Adulto, Alfabetización en Salud, Promoción de la Salud, Estudio de Validación, Encuestas y Cuestionarios, Sistema Único de Salud, Adulto

## Abstract

to investigate the psychometric properties of the Brazilian Portuguese version of the health literacy questionnaire European Health Literacy Survey Questionnaire short-short form (HLS-EU-Q6) in Brazilian adults.

**Method:**

the instrument was translated and pre-tested in a sample of 50 individuals. Subsequently, it was applied to a sample of 783 adult individuals. The data went through an appropriate process of testing the properties, with the combination of techniques of Exploratory Factor Analysis, Confirmatory Factor Analysis and Item Response Theory. For the assessment of reliability, the Cronbach's alpha and McDonald's Omega indicators were used. Cross-validation with full data analysis was applied.

**Results:**

the majority of the participants was female (68.1%), with a mean age of 38.6 (sd=14.5) years old and 33.5% studied up to elementary school. The results indicated a unidimensional model with an explained variance of 71.23%, adequate factor load levels, commonality and item discrimination, as well as stability and replicability of the instrument to other populations.

**Conclusion:**

the Brazilian version of HLS-EU-Q6 indicated that the instrument is suitable for indiscriminate application in the population to which it is intended to assess health literacy levels.

## Objetivo

investigar as propriedades psicométricas da versão adaptada ao português do Brasil do questionário de letramento em saúde *European Health Literacy Survey Questionnaire short-short form* (HLS-EU-Q6) em adultos brasileiros.

**Método:**

o instrumento foi traduzido e pré-testado em uma amostra de 50 indivíduos. Em seguida, ele foi aplicado em uma amostra com 783 indivíduos adultos. Os dados passaram por um processo adequado de testagem das propriedades, com a combinação de técnicas da Análise Fatorial Exploratória, Análise Fatorial Confirmatória e Teoria de Resposta ao Item. Para a avaliação da confiabilidade, foram utilizados os indicadores de alfa de Cronbach e o Ômega de McDonald. Foi aplicada a validação cruzada com análise integral dos dados.

**Resultados:**

a maioria dos participantes era do sexo feminino (68,1%), com idade média de 38,6 (dp=14,5) anos e 33,5% estudaram até o ensino fundamental. Os resultados indicaram um modelo unidimensional com variância explicada de 71,23%, níveis de cargas fatoriais, comunalidades e discriminação do item adequados, bem como estabilidade e a replicabilidade do instrumento para outras populações.

**Conclusão:**

a versão brasileira do HLS-EU-Q6 indicou que o instrumento está adequado para aplicação de forma indiscriminada na população a que se destina para aferir os níveis de letramento em saúde.

## Abstract (es)

**Objetivo:**

investigar las propiedades psicométricas de la versión en portugués brasileño del cuestionario de alfabetización en salud *European Health Literacy Survey Questionnaire short-short form* (HLS-EU-Q6) en adultos brasileños.

**Método:**

el instrumento fue traducido y probado primero en una muestra de 50 individuos. Luego, se aplicó a una muestra de 783 individuos adultos. Los datos pasaron por un proceso adecuado de prueba de las propiedades, con la combinación de técnicas de Análisis Factorial Exploratorio, Análisis Factorial Confirmatorio y Teoría de Respuesta al Ítem. Para evaluar la confiabilidad se utilizaron los indicadores alfa de Cronbach y Omega de McDonald's. Se aplicó validación cruzada con análisis de datos completo.

**Resultados:**

la mayoría de los participantes era de sexo femenino (68,1%), con una edad media de 38,6 (dt = 14,5) años y el 33,5% cursó hasta la educación primaria. Los resultados indicaron un modelo unidimensional con una varianza explicada de 71,23%, niveles adecuados de carga factorial, comunalidades y discriminación de ítems, así como estabilidad y replicabilidad del instrumento a otras poblaciones.

**Conclusión:**

la versión brasileña de HLS-EU-Q6 indicó que el instrumento es apto para su aplicación indiscriminada en la población a la que se destina para evaluar los niveles de alfabetización en salud.

## Introduction

Health Literacy (HL) is a construct related to the use of multiple forms of health information in the most varied contexts^(^ 1 ^)^. Although there are several definitions, which include personal characteristics, social resources and the role of the health services in this process^(^ 1 ^-^ 2 ^)^, for the World Health Organization (WHO), HL concerns the knowledge, motivations and skills of people to access, understand, judge and apply health information, in order to make decisions that help them navigate the health systems, as well as promote, prevent and care for their health^(^ 1 ^)^.

A number of studies indicate associations between individuals with low levels of HL and less participation in activities that promote health and related to disease prevention, less assertive health choices, worse self-control of chronic diseases, higher frequency of hospitalizations and cases of morbidity and mortality, with a consequent increase in costs for the health systems^(^ 1 ^,^ 3 ^)^. In view of this, HL is considered by the WHO as an important social determinant of health, influenced by socioeconomic and cultural characteristics and by the functioning of the health systems^(^ 1 ^)^.

Several instruments have already been developed to measure this construct in individuals and populations^(^ 4 ^-^ 5 ^)^; however, most assess only the functional characteristics of HL, that is, the personal skills to read and understand written and oral health-related information^(^ 1 ^,^ 6 ^)^. In order to overcome this gap, a European consortium of research institutions developed a multidimensional and integrative model of the HL and developed an instrument for its measurement consisting of 47 items, called HLS-EU-Q47^(^ 1 ^,^ 7 ^)^. The HLS-EU-Q47 questionnaire assesses individual skills in understanding, evaluating and applying health-related information and was developed based on a conceptual literacy model that integrates three domains: health care (16 questions), health promotion (16 questions) and disease prevention (15 questions). Its answer options are arranged on a four-point Likert scale that ranges from 1 for very difficult to 4 for very easy^(^ 7 ^-^ 9 ^)^. As it takes nearly 10 minutes to fill in, shorter versions have been developed, that is, HLS-EU-Q16 (short form) and HLS-EU-Q6 (short short form), which present 16 and six questions, respectively^(^ 9 ^)^. However, so far, few studies have used HLS-EU-Q6^(^ 7 ^,^ 9 ^-^ 12 ^)^ and/or evaluated its psychometric properties^(^ 13 ^-^ 14 ^)^, showing the importance of testing it more robustly in other populations.

Although most of the research studies on HL are concentrated in the European continent, North America and Australia^(^ 1 ^-^ 2 ^)^, there has been an expansion of studies in other parts of the world in the last decade, as in Brazil^(^ 15 ^-^ 16 ^)^, including the creation of the Brazilian Health Literacy Network (Rede Brasileira de Letramento em Saúde, REBRALS). Bearing in mind the low level of schooling and the difficulties in understanding the professional recommendations by the Brazilian population^(^ 15 ^-^ 16 ^)^, it is important that there are simple and short instruments to measure the construct of HL in this context, in order to make it applicable in the practice of the services.

In our country, the term literacy has been translated as alfabetização, literacia and letramento^(^ 17 ^)^. However^(^ 17 ^)^, although both are inseparable processes, alfabetização must be understood as the "process of acquisition and appropriation of the writing, alphabetical and orthographic system" while literacy as "the development of practical skills of reading and writing in social practices involving the written language, and of positive attitudes in relation to these practices".

Despite this fruitful research context, it is also noted that, to date, few instruments have been validated to measure HL in the Brazilian population that add broader aspects of the construct to, in addition to measuring its functional aspects, that are easy and quick to apply^(^ 18 ^)^. Thus, the aim of the present study was to analyze the evidence of the psychometric properties of the HLS-EU-Q6 instrument, validated for Brazilian Portuguese.

## Method

The research project was submitted to and approved by the Research Ethics Committee (CAAE: 58131216.5.0000.5418). Initially, Professor Kristine Sørensen, the author responsible for the instrument, was asked to authorize its translation into Brazilian Portuguese.

The instrument was translated and adapted according to the literature recommendations^(^ 19 ^-^ 20 ^)^. To this end, the original version of the HLS-EU-Q47 questionnaire was translated from English into Brazilian Portuguese by two English teachers and a health researcher with knowledge of the English language.

The consensus version was then back-translated into English (back-translation) by two native English-speaking translators who did not participate in the first stage of the translation. A committee of experts, composed of six experts in the health area with experience in the field of Literacy in Health and with a high level of proficiency in English, as well as a Portuguese teacher and a Linguistics professor, evaluated the entire process of translation and back-translation and proposed a final version of the instrument, the English version being submitted for evaluation and approval by the responsible author. The version of HLS-EU-Q47 was then applied to 50 adult individuals, users of the health services in the municipalities of Piracicaba/SP; São Paulo/SP, Aparecida de Goiânia/GO and Fortaleza/CE, randomly selected. There was no need for changes in the instrument after this pre-test phase.

The evaluation of the psychometric properties of HLS-EU-Q6 was carried out through a cross-sectional study with a sample of 783 adult individuals. Of them, 320 lived in an area assigned to a Basic Health Unit (BHU) located in the city of São Paulo/SP and 293 lived in areas close to three Family Health Units (FHUs) located in the city of Piracicaba/SP. The residences were drawn at random. With the registration of users, and with the help of a cell phone program, the individuals were invited to participate in the research and interviewed at their homes. In addition, 50 individuals accompanying a reference institution for cancer treatment in Fortaleza/CE participated in the study, who were waiting in the waiting room and were randomly invited to participate in the research. Finally, 120 users of an FHU located in Aparecida de Goiânia/GO who were waiting for assistance in the waiting room and accepted to participate in the research were randomly invited to participate.

To calculate the sample size, a proportion of at least 15 adults was considered for each question in the questionnaire, higher than the general recommendation of 10:1 found in the literature, which allows for more accurate analyses^(^ 21 ^)^.

The HLS-EU-Q6 questionnaire is called short-short form and consists of six questions from HLS-EU-Q47^(^ 7 ^,^ 9 ^)^ [On a scale that goes from "very easy" to "very difficult", how easily you can: 1. assess when you need a second opinion from another doctor?; 2. use the information that your doctor gives you to make decisions about your illness?; 3. find information on how to deal with mental health problems, such as stress or depression?; 4. assess whether the information on health risks available in the media is reliable? (e.g. TV, Internet or other means of communication); 5. find information about activities that are good for your mental well-being? (e.g. meditation, exercise, walking, pilates, etc.); 6. understand the information available in the media on how to stay healthier? (e.g. Internet, newspapers, magazines)]. Questions 1 and 2 are related to the evaluation and application of diverse information relevant to health in the field of health care, while questions 3 and 4 deal with finding/accessing and evaluating information in the field of disease prevention. Finally, questions 5 and 6 investigate the individual's ability to find/access and understand information relevant to health in the field of health promotion^(^ 7 ^,^ 9 ^)^.

The final individual score is a mean calculated by summing up the answers to the six questions divided by the number of items answered. The score is calculated as long as at least five of the six questions are answered differently from 1, and varies between 1 and 4, with higher values indicating better levels of HL. According to the authors of the instrument, the final score values classify individuals according to three levels of HL: inadequate (≤ 2); problematic (> 2 and ≤ 3); and sufficient (> 3)^(^ 7 ^,^ 9 ^-^ 10 ^)^.

For statistical analysis, data went through an extensive and robust process of testing the properties, with the combination of techniques of Exploratory Factor Analysis (EFA), Confirmatory Factor Analysis (CFA) and Item Response Theory (IRT), aiming at searching for strong evidence of validation in the construction stage and its stability for other subsamples. EFA requires the fulfillment of several stages, such as: data inspection techniques, the factor analysis method, the retention and rotation technique and the factor quality indexes^(^ 22 ^)^.

Dimensionality testing was performed by Robust Parallel Analysis using Optimal implementation of Parallel Analysis with a minimun rank factor analysis that minimizes the common variance of the residuals^(^ 23 ^)^. The robustness of the test was determined by associating a bootstrap with a sample extrapolation to 5,000. The estimation of the polychoric matrix was performed using the Bayes Modal Estimation^(^ 24 ^)^.

Dimensionality, in the exploratory factor analysis (unrestricted model), was tested by Parallel Analysis, which has been considered one of the most effective and accurate techniques for testing the number of factors/dimensionality^(^ 25 ^-^ 27 ^)^. The factors were extracted using the RULS (Robust Unweighted Least Squares) technique, which reduces the residuals of the matrices^(^ 27 ^)^.

As a complementary analysis to test the number of factors, the following techniques of unidimensionality/multidimensionality were applied^(^ 28 ^)^: UNICO (Unidimensional Congruence >0.95), ECV (Explained Common Variance >0.80 - QUINN, 2014) and MIREAL (Mean of Item Residual Absolute Loadings <0.30). These techniques were applied to the instrument and the items. In the case of the items, they were used to guarantee and assess whether the item would adhere in a unidimensional or multidimensional manner, that is, if there was a possibility that the item would load significantly in more than one dimension. The explained variance of the instrument should be around 60% and the initial factorial loads around 0.30^(^ 22 ^)^. In addition, mean commonality values between 0.40 and 0.60 are found^(^ 29 ^)^. The maintenance or removal of an item from the model will depend on the magnitude of the commonality, the factor loads, the sample size and the degree with which the item can measure the factor and the absence of cross-loading.

To confirm the adjustment of the factorial loads, the Normal-Ogive Graded Response Model^(^ 30 ^)^ technique was used for polytomous structure, by means of the Item Response Theory. The discrimination index of the item (a) was adopted, which measures the association strength between the item and the latent variable and has a similar interpretation to the factorial loads of the exploratory factor analysis^(^ 31 ^)^ to complement it. Baker's recommendation^(^ 32 ^)^ was adopted that "a" <0.65 is considered to have low discrimination power; between 0.65 and 1.34, moderate discrimination, between 1.35 and 1.69, high discrimination; and above 1.70, very high discrimination.

For the CFA adjustment indices, factor loads greater than 0.50 and the following minimum indices for adequacy were considered, considering the number of participants and variables: NNFI (Non-Normed Fit Index >0.95); CFI (Comparative Fit Index >0.95); GFI (Goodness Fit Index >0.95); AGFI (Adjusted Goodness Fit Index >0.95); RMSEA (Root Mean Square Error of Approximation <0.08) and RMSR (Root Mean Square of Residuals <0.08)(22).

The reliability of the instrument was assessed using two indicators: Alfa^(^ 33 ^)^ and Omega^(^ 34 ^)^. The adoption of two indicators sought to increase the reliability of the interpretation, as numerous reliability inconsistencies have been reported through Cronbach's alpha^(^ 35 ^-^ 36 ^)^.

The replicability of the construct was assessed by the Generalized G-H Index^(^ 37 ^)^ with an index greater than 0.80(28) and, for the quality and effectiveness of the factor estimation, the Factor Determinacy Index was used, pointing for an adequate estimate values greater than 0.90, EAP marginal reliability (>0.80), sensibility ratio (SR >2) and Expected percentage of true differences (EPTD >90%). The application of multiple indicators stems from the need to certify the instrument's validity evidences by various techniques. In addition, the application and interpretation of the model's adjustment indexes (Goodness-Of-Fit - GOF), by themselves, do not guarantee that the factor analysis solution is good or useful in the practice, as it is possible to obtain satisfactory solution rates based on low quality items^(^ 38 ^-^ 39 ^)^.

In order to increase the reliability and replicability of the proposed model^(^ 40 ^)^,cross-validation was applied, as well as the Holdout technique^(^ 41 ^)^. This technique divides the bank into a training sample that can vary between 10%, 30% and 50% and another set of data, called the test bank^(^ 41 ^)^. The database was divided 50/50 with random selection of items. The Random.org website (www.random.org) and the random sequence generator technique were used to divide the groups. The banks were named as follows: complete sample (CS with 783 cases); sample 1 (S1 - training bench with 392 cases) and sample 2 (S2 - test bench with 391 cases). Another modification is that, usually, in cases of application of EFA and CFA, the tendency is to use the first training bank in EFA and the test bank in CFA(22). In this study, it was decided to apply the analysis procedures in order to expand the evidence of validity and the quality of the instrument. The analysis was extended to the complete sample (CS) if the adjustment occurred in the two samples. The analyses started from the training bank (sample 1) and, immediately after, in the other two data sets for each set of techniques.

The analyses were performed using SPSS 23, AMOS 23 and Factor 10.8.01.

## Results

A total of 783 individuals participated in the study, with a mean age of 38.6 (14.5) years old, of which 68.1% (n=533) were female. In addition, 262 (33.5%) studied until elementary school and 52.7% used the public health system as the only way to access the health services. Regarding the characteristics of the sub-samples, 32.5%, 33.7%, 38% and 39% of the participants were male, respectively, in Piracicaba, São Paulo, Aparecida de Goiânia and Fortaleza. The mean age of the participants was 41.6; 39.6; 38.2 and 38.1 years old, also in that order, in Piracicaba, São Paulo, Aparecida de Goiânia and Fortaleza. Regarding the schooling level, 82.6%; 78.3%; 78% and 81.6% of the participants had completed high school, respectively, in Piracicaba, São Paulo, Aparecida de Goiânia and Fortaleza. Of the participants, 52.7% used the public health system as the only way to access the health services.

Only 91 (1.9%) of the 4,698 possible answers were missing and the software (Factor) itself simulates the effects of the missings to correct the model^(^ 42 ^)^.

Regarding the psychometric analyses of the HLS-EU-Q6 instrument, the sample adequacy indices based on polychoric correlation indicated good levels of factorability for the three bank configurations. Sample 1: Kaiser-Meyer-Olkin index (KMO = 0.82), Bartlett's sphericity = 314.5 (df = 15; P <0.0001) and the matrix determinant = 0.19 (<0.0001). Sample 2: KMO = 0.82, Bartlett's sphericity = 342.8 (df = 15; P <0.0001) and the matrix determinant = 0.17. For the Complete Sample, KMO was 0.84, Bartlett's sphericity = 636.6 (df = 15; p <0.0001) and the matrix determinant = 0.19 (p <0.0001).

The first analysis was centered on the study of the instrument's dimensionality/factors and the parallel analysis (PA) indicated the existence of only one dimension for the instrument with an explained variance of 69.92% of the latent variable, above the recommended minimum in initial models(34). The eigenvalues also pointed to only one dimension with an eigenvalue of 3.62. There was no indication that this set of items could be aligned in a multidimensional model.

In sample 2, the PA indicated the existence of only one dimension for the instrument with an explained variance of 68.95%. The same unidimensionality occurred using the eigenvalue criterion (3.76). The analysis with the complete database demonstrated the unidimensionality by the AP with an explained variance of 71.23%, and the same occurred by the Kaiser criteria (eigenvalue = 3.69). Unidimensionality was confirmed by the values of Unico (S1 = 0.98; S2 = 0.98 and CS = 0.99), for ECV (S1 = 0.86; S2 = 0.87 and CS = 0.89) and MIREAL (S1 = 0.24; S2 = 0.29 and CS = 0.23). As an extensive way of testing unidimensionality, the indices were applied to the items and the results can be seen in Table 1.

**Table 1:** Values of UNICO, ECV and MIREAL of the items of the Brazilian version of the HLS-EU-Q6 instrument for the three samples analyzed. Piracicaba/SP; São Paulo/SP, Aparecida de Goiânia/GO and Fortaleza/CE, Brazil, 2018

ITEM	UNICO*			ECV^†^			MIREAL^‡^		
	Sample 1	Sample 2	Complete Sample	Sample 1	Sample 2	Complete Sample	Sample 1	Sample 2	Complete Sample
ITEM 1	0.93	0.98	0.98	0.72	0.83	0.85	0.52	0.28	0.30
ITEM 2	0.99	0.99	0.98	0.95	0.87	0.86	0.15	0.33	0.29
ITEM 3	1.00	0.98	0.99	0.99	0.86	0.96	0.05	0.28	0.13
ITEM 4	0.99	0.99	1.00	0.92	0.91	0.98	0.21	0.21	0.10
ITEM 5	0.99	0.98	0.99	0.93	0.85	0.90	0.17	0.30	0.22
ITEM 6	0.98	0.99	0.98	0.84	0.88	0.85	0.35	0.32	0.35

* UNICO = Unidimensional Congruence;

† ECV = Explained Common Variance;

‡ MIREAL = Mean of item residual absolute loadings

The results of the dimensionality of the items indicated that all the items in all the samples presented the unidimensional I-UNICO. The I-ECV showed a small violation of item 1 for S1, with all other unidimensional items.

In I-REAL there was a violation of item 1 in S1 and of item 6, marginally, in the three samples. In the CS, only item 6 showed the residuals of the factorial load slightly above 0.30. Again, the indicators predominantly pointed to unidimensionality, with no breach of this in this first phase of the analysis.

The fact that the instrument is unidimensional did not require the use of rotational techniques of the factorial matrix and indicated the application of the Normal-ogive graded response model technique in the IRT, suitable for the polytomous unidimensional model.

Table 2 shows the values of the factorial loads, commonality and item breakdown for the three samples.

**Table 2:** Factorial loads, commonality and item breakdown for the Brazilian version of the HLS-EU-Q6 instrument. Piracicaba/SP; São Paulo/SP, Aparecida de Goiânia/GO and Fortaleza/CE, Brazil, 2018

ITEM	Factorial load (λ)			Commonalities (h2)			Item breakdown (a)		
	Sample 1	Sample 2	Complete Sample	Sample 1	Sample 2	Complete Sample	Sample 1	Sample 2	Complete Sample
ITEM 1	0.76	0.64	0.72	0.58	0.41	0.52	1.17	0.83	1.04
ITEM 2	0.67	0.83	0.72	0.45	0.68	0.52	0.91	1.48	1.04
ITEM 3	0.67	0.68	0.70	0.45	0.47	0.49	0.91	0.94	0.99
ITEM 4	0.75	0.72	0.75	0.56	0.52	0.56	1.13	1.04	1.14
ITEM 5	0.68	0.72	0.68	0.46	0.52	0.46	0.92	1.04	0.92
ITEM 6	0.80	0.84	0.83	0.64	0.72	0.67	1.34	1.61	1.44

The factorial loads were established between 0.64 and 0.84 in the samples, which indicates satisfactory and adequate levels. No collinearity/multicollinearity problems and Heywood Cases were found. When factorial loads are above 0.85, collinearity/multicollinearity can indicate redundancy of the items and problems with data distribution and generate distortions in the measurement of the latent variable. Likewise, no violations of the factor load limit (-1 to +1) were found. This type of violation is called Heywood Cases and is an indicator of possible sample inadequacies, improper estimates of the error variance and model uncertainty. The absence of these problems allows us to assert that the solution of the model was appropriate and that there were no deleterious effects arising from the sample and, mainly, from the established model.

All the commonalities were above 0.40, with a range between 0.41 and 0.72. All the items presented discrimination values between 0.83 and 1.61, ranging from moderate to high discrimination. Thus, the factorial loads (λ), commonalities (h2) and item breakdown (a) presented adequate and consistent levels for the unidimensional model.

The values for the reliability indicators for S1 for Cronbach's alpha and McDonald's omega were 0.86. For S2 it was 0.87 for alpha and 0.88 for omega. In the CS, it was 0.87 for both indices.

As for the replicability of the construct by the latent and observed G-H index, the scores were 0.87, 0.89 and 0.88 for latent G-H, respectively, for samples S1, S2 and CS. The G-H observed was 0.78, 0.77 and 0.79, also in due order, for samples S1, S2 and CS. As there was a small difference between the levels of latent and observed G-H, stability of the model can be inferred even when applied to other population samples and its consequent generalization.

For the measures of quality and effectiveness of the model scores , the FDI presented a high and adequate level (S1 = 0.93; S2 = 0.95 and CS = 0.88) to assess the relationship between the estimation of the solution scores and the latent variable to which they estimate. EAP (S1 = 0.87; S2 = 0.89 and CS = 0.93), SR (S1 = 2.64; S2 = 2.92 and CS = 2.72), and EPTD (S1 = 91.4%; S2 = 92.3% and CS = 91.6%) also indicated quality and effectiveness of the model solution.

The Exploratory Factor Analysis and the IRT indices pointed to a unidimensional model consistent with maintaining satisfactory levels in all stages of analyses.

**Figure 1:**
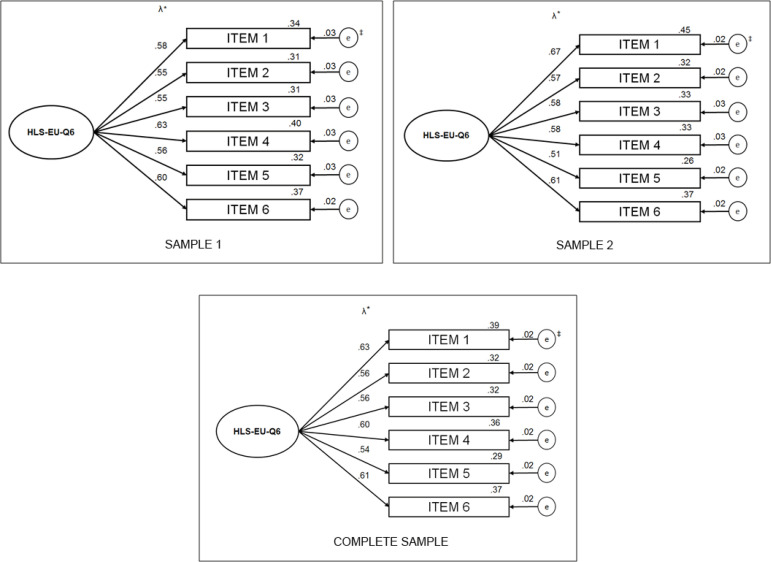
Path diagram for each of the samples *Factorial load; ‡Standard error

About the CFA, the path diagram has been established for each of the samples. Figure 1 shows the results of the factorial loads, predictive power of the item (R2) and standard error for S1, S2 and complete model (Figure 1).

S1's CFA presented items with factorial loads varying between 0.55 and 0.63, that is, above the recommended minimum of 0.50. The predictive values of the R2 items were established between 0.31 and 0.40. The path diagram of sample 2 showed levels similar to that of model 1 (S1) for the evaluated indicators. The factorial loads ranged from 0.51 to 067 with predictive values of the items ranging from 0.26 to 0.45, therefore demonstrating satisfactory levels. The path diagram for the complete sample also established a model with adequate levels for the factor loads, which ranged from 0.54 to 0.63 and with item prediction levels from 0.29 to 0.37. This fact indicated stable, satisfactory and consistent results for the three samples tested.

Table 3 shows the values of the eigenvalues by the correlation and covariance, factorial loads, item prediction level (R2), residuals and standard error for the three samples.

**Table 3:** Eigenvalues, factorial loads, R2^‡^, residuals and standard error of the models evaluated for the Brazilian version of the HLS-EU-Q6 instrument for the three samples evaluated. Piracicaba/SP; São Paulo/SP; Aparecida de Goiânia/GO and Fortaleza/CE, Brazil, 2018

Indices	Sample 1	Sample 2	Complete Sample
Eigenvalue (r)*	2.70	2.72	2.70
Eigenvalue (cov)^†^	1.11	1.14	1.12
Factorial loads	0.55 to 0.60	0.51 to 0.67	0.53 to 0.64
R2^‡^	0.30 to 0.37	0.26 to 0.45	0.28 to 0.41
Residuals	-0.018 to 0.019	-0.033 to 0.051	-0.021 a 0.022
Standard error	0.029 a 0.036	0.019 a 0.030	0.021 a 0.026

* r = Eigenvalues by the correlation;

† cov = Eigenvalues by the variance;

‡ R2 = Item prediction level

It is essential to point out that, in the three samples tested in the CFA, both the calculation of eigenvalues by correlation and covariance indicated that the models are unidimensional.

It was verified, through the concept of correlation, that the eigenvalues were 2.70, 2.72 and 2.70, respectively for S1, S2 and CS. In addition, due to the covariance concept, the values were 1.11, 1.14 and 1.12 for the three samples. There were no other eigenvalues above 1.

Table 4 shows all the global indices of EFA, CFA, IRT, reliability, replicability, quality and effectiveness of the model. All the indicators analyzed pointed to a unidimensional, consistent, accurate and stable model.

**Table 4:** Synthesis of the model for the Brazilian version of the HLS-EU-Q6 instrument. Piracicaba/SP; São Paulo/SP; Aparecida de Goiânia/GO and Fortaleza/CE, Brazil, 2018

	Index	Technique	Sample 1	Sample 2	Complete Sample
Exploratory	Adequacy of correlation matrix	Determinant of the matrix Bartlett	0.19	0.17	0.19
			314.0 (df = 15)	342.8 (df = 15)	636.3 (df = 15)
		KMO (Kaiser-Meyer-Olkin)	0.82	0.82	0.84
	Explained Variance (Kaiser Criterion)		60.39%	62.68%	61.63%
	Explained Variance (AP)		69.92%	68.95%	71.23%
	Polychoric Correlation (rp = )		0.39 a 0.68	0.37 a 0.69	0.40 a 0.65
	Robust Mean-Scaled Chi Square (X2/df = 56)		20.19 (df = 9)*	22.69 (df = 9)*	22.54 (df = 9)*
Confirmatory	Non-Normed Fit Index (NNFI)		0.97	0.97	0.98
	Comparative Fit Index (CFI)		0.98	0.98	0.99
	Goodness of Fit Index (GFI)		0.99	0.98	0.99
	Adjusted Goodness of Fit Index (AGFI)		0.98	0.97	0.99
	Root Mean Square Error of Approximation (RMSEA)		0.08	0.08	0.06
Reliability	Root Mean Square of Residuals (RMSR)		0.05	0.08	0.05
	Standardized Cronbach's Alpha		0.86	0.87	0.87
	McDonald's Omega		0.86	0.88	0.87
	Construct Reliability - Index G H (Latente e observada)		(0.87; 0.78)	(0.89; 0.77)	(0.88; 0.79)
Unidimensionality	Unidimensional Congruence (UNICO)		0.98	0.98	0.99
	Explained Common Variance (ECV)		0.86	0.87	0.89
	Mean of item residual absolute loading (MIREAL)		0.24	0.29	0.23
Quality and effectiveness	Factor Determinacy Index (FDI)		0.93	0.94	0.88
	EAP Marginal Reliability		0.87	0.89	0.93
	Sensivity Ratio (SR)		2.64	2.92	2.72
	Expected percentage of true differences (EPTD)		91.4%	92.3%	91.6%

According to the results of the HLS-EU-Q6 scores, only 2% of the participants were classified as having sufficient levels of HL; 51.7% with problematic levels, and 46.3% with inadequate levels.

## Discussion

The results of the present study demonstrated that the Brazilian Portuguese version of HLS-EU-Q6 showed unidimensional characteristics, satisfactory factor loads and good levels of reliability, which point to an instrument with evidence of a consistent and reliable internal structure for measuring the desired construct.

To date, this is the first study to assess the validity of HLS-EU-Q6 using multiple sizing techniques and model adjustment indices. In the European Health Literacy Study (HLS-EU), the adjustment of the model to HLS-EU-Q6 was assessed by the CFA in subsamples that responded to the complete instrument, and a satisfactory factor structure was observed in the most samples from participating countries^(^ 7 ^,^ 9 ^)^. The Brazilian version of HLS-EU-Q6, on the other hand, demonstrated characteristics of unidimensionality and good adjustment in EFA, IRT and CFA in all samples evaluated in cross-validation. The psychometric techniques applied in the study are much more extensive and contemporary than in studies in other countries.

The analyses also bring a series of indicators that are rare to be carried out in psychometric studies, some because they are recent, and are not available in commercial software programs, and the use of more extensive data analysis techniques, which incorporate the concept of evidence of validity of the external structure by multiple indicators. There has been progress in recent years in the expansion of multiple techniques. As they point out^(^ 22 ^)^, few studies applied multiple techniques for validation analysis. Therefore, there is a substantial advance, but still insufficient in this practice, especially when many studies still use the Kaiser and Scree-Test criteria as a criterion for retaining the model. Another factor is the application of a complete cross-validation, instead of partial, when using the training bench in EFA and the test bench in CFA, this in order that possible errors existing in the analysis of the EFA are transposed to the CFA. It is understood for those who do not have deep knowledge of psychometry that CFA (restricted model) is superior to EFA (unrestricted model). It should be made clear that CFA may also not lead to adjustment, due to model inaccuracy, error in the number of factors, omission of cross-loading and correlation errors^(^ 43 ^)^. The complete application of the technique, similar to what is done in the K-folds, ensures that the model, due to the subsamples, can be extrapolated to more heterogeneous populations. In addition, the application of the G-H Index assesses how well-defined the latent variable is from the instrument items, that is, the viability of a measurement model given by a set of items. Such analyses make it possible to assess the probability of the model being stable across studies, populations or subpopulations^(^ 28 ^,^ 44 ^)^. Thus, even in the sample of this study, composed of individuals from four cities, there was no instability in the instrument, ensuring the quality, effectiveness, stability and replicability of the final model in different contexts. In addition, all IRT indicators in the three samples were established at adequate levels, reinforcing and legitimizing the results obtained in the primary indicators of factor analysis. These indicators are part of a set of analyses that attest to the reliability of the instrument.

In this same way, regarding the reliability of the instrument, the alpha values found for the three samples of the Brazilian version showed good values, and higher than those found in the total sample of the European study (α=0.803)(7.9), in adults in Italy (α=0.672)^(^ 13 ^)^ and in France (α=0.83)^(^ 14 ^)^, as well as a study with diabetics in Belgium (α=0.797)^(^ 12 ^)^. Furthermore, the omega values corroborated the reliability of the Brazilian version of the instrument.

Among the HL dimensions that can be assessed with the Brazilian version of HLS-EU-Q6 are the following: the evaluation and application of general health information; finding, accessing and evaluating information for disease prevention and health promotion^(^ 7 ^,^ 9 ^)^. The application of instruments for tracking HL skills in the reception in the health services has been recommended to qualify the data collection of users and as a means for the health professionals, including nurses, to guide their care, being considered as the "sixth vital sign"^(^ 1 ^,^ 45 ^)^.

The percentage of individuals with inadequate HL levels in the present study (46.3%) was higher than the mean found in the countries of the European study (9%), as well as in France (5%), Italy (8.9%) and Belgium (9.8%)(7.9,12-14). This may have occurred due to different socioeconomic characteristics of the populations analyzed, since the individuals' schooling and income levels in the aforementioned studies were much higher than those of the present study. In addition, the cognitive, cultural, organizational characteristics of the educational and health system may have contributed to these differences^(^ 7 ^,^ 9 ^,^ 13 ^)^. Although it is not the objective of this study, the identification that only 2% of the participants had sufficient levels of HL, that is, they were able to find, access, understand, evaluate and use the health information indicated for the importance of the professionals knowing which individual limitations directly impact on health care.

The present study brings important advances in the scientific knowledge related to the validation process of instruments for the measurement of HL and, also, the availability of an instrument with extensive evidence of validity to assess the HL of the Brazilian population with quick and easy application, a fact that will enable its insertion in the routine of the health services.

The measurement of HL by means of HLS-EU-Q6 can help the health professionals to redirect the interventions in the area in order to identify the real needs of the users of the health services, making it a new possibility to think and execute patient-centered care. The WHO recommends that quantifying the limitations related to HL is an important step towards such an action.

Thus, it is recommended that future studies expand the application of this instrument to other samples and populations, aiming at knowing the classification of the literacy levels (inadequate, problematic and sufficient) for the different regions of the country.

## Conclusion

The Brazilian version of the HLS-EU-Q6 instrument indicated diverse evidence of adequate internal structure validity for measuring the health literacy levels of Brazilian adults. Therefore, it is a tool that can be easily used in the clinical practice, capable of quickly and objectively measuring the limitations in access, understanding and use of health information, whether for disease prevention or for health promotion.

## References

[1] Okan O, Bauer U, Levin-Zamir D, Pinheiro P, Sorensen K (2019). International Handbook of Health Literacy: Research, practice and policy
across the lifespan..

[2] Liu C, Wang D, Liu C, Jiang J, Wang X, Chen H (2020). What is the meaning of health literacy? A systematic review and
qualitative synthesis. Fam Med Community Health.

[3] Palumbo R (2017). Examining the impacts of health literacy on healthcare costs. An
evidence synthesis. Health Serv Manage Res.

[4] Liu H, Zeng H, Shen Y, Zhang F, Sharma M, Lai W (2018). Assessment Tools for Health Literacy among the General
Population: A Systematic Review. Int J Environ Res Public Health.

[5] Nguyen TH, Paasche-Orlow MK, McCormack LA (2017). The State of the Science of Health Literacy
Measurement. Stud Health Technol Inform.

[6] Nutbeam D, McGill B, Premkumar P (2018). Improving health literacy in community populations: a review of
progress. Health Promot Int.

[7] Pelikan JM, Ganahl K, Logan RA, Siegel ER (2017). Measuring Health Literacy in General Populations: Primary
Findings from the HLS-EU Consortium's Health Literacy Assessment
Effort. Health Literacy: New Directions in Research, Theory and
Practice.

[8] Duong TV, Aringazina A, Baisunova G, Nurjanah, Pham TV, Pham KM (2017). Measuring health literacy in Asia: Validation of the HLS-EU-Q47
survey tool in six Asian countries. J Epidemiol.

[9] Pelikan JM, Röthlin F, Ganahl K, Boltzmann L (2014). Measuring comprehensive health literacy in general populations - the
HLS-EU instruments.

[10] Amoah PA, Philllips DR, Gyasi RM, Koduah AO, Edusei J (2017). Health literacy and self-perceived health status among street
youth in Kumasi, Ghana. Cogent Med.

[11] Vandenbosch J, den Broucke SV, Schinckus L, Schwarz P, Doyle G, Pelikan J (2018). The impact of health literacy on diabetes self-management
education. Health Educ J.

[12] Schinckus L, Dangoisse F, Van den Broucke S, Mikolajczak M (2018). When knowing is not enough: Emotional distress and depression
reduce the positive effects of health literacy on diabetes
self-management. Patient Educ Couns.

[13] Lorini C, Lastrucci V, Mantwill S, Vettori V, Bonaccorsi G, Florence Health Literacy Research Group (2019). Measuring health literacy in Italy: a validation study of the
HLS-EU-Q16 and of the HLS-EU-Q6 in Italian language, conducted in Florence
and its surroundings. Ann Ist Super Sanita.

[14] Rouquette A, Nadot T, Labitrie P, Van den Broucke S, Mancini J, Rigal L (2018). Validity and measurement invariance across sex, age, and
education level of the French short versions of the European Health Literacy
Survey Questionnaire. PLoS One.

[15] Rigolin CCD, Bastos JC, Mello LC, Carvalho CCB (2018). The Brazilian scientific production of theses and dissertations
on health literacy. R Tecnol Soc.

[16] Maragno CAD, Mengue SS, Moraes CG, Rebelo MVD, Guimarães AMM, Pizzol TDSD (2019). Test of health Literacy for Portuguese-speaking
Adults. Rev Bras Epidemiol.

[17] Soares M (2004). Multiple facets of literacy and initial reading
instruction. Rev Bras Educ.

[18] Health Literacy Tool Shed.

[19] Beaton DE, Bombardier C, Guillemin F, Ferraz MB (2001). Guidelines for the process of cross-cultural adaptation of
self-report measures. Spine.

[20] Reichnheim ME, Moraes CL (2007). Operationalizing the cross-cultural adaptation of epidemological
measurement instruments. Rev Saúde Pública.

[21] Hair JR, Black WC, Babin BJ, Anderson R., Tatham RL (2018). Multivariate data analysis.

[22] Goretzko D, Pham TTH, Bühner M (2019). Exploratory factor analysis: Current use, methodological
developments and recommendations for good practice. Curr Psychol.

[23] Timmerman ME, Lorenzo-Seva U (2011). Dimensionality Assessment of Ordered Polytomous Items with
Parallel Analysis. Psychol Methods.

[24] Choi J, Kim S, Chen J, Dannels S (2011). A comparison of maximum likelihood and Bayesian estimation for
polychoric correlation using Monte Carlo simulation. J Educ Behav Stat.

[25] Finch WH (2020). Using Fit Statistic Differences to Determine the Optimal Number
of Factors to Retain in an Exploratory Factor Analysis. Educ Psychol Meas.

[26] Dobriban E, Owen AB (2019). Deterministic Parallel Analysis An Improved Method for Selecting
Factors and Principal Components. J Royal Stat Soc-B.

[27] Auerswald M, Moshagen M (2019). How to determine the number of factors to retain in exploratory
factor analysis: A comparison of extraction methods under realistic
conditions. Psychol Methods.

[28] Ferrando PJ, Lorenzo-Seva U (2018). Assessing the quality and appropriateness of factor solutions and
factor score estimates in exploratory item factor analysis. Educ Psychol Meas.

[29] Hattori M, Zhang G, Preacher KJ (2017). Multiple Local Solutions and Geomin Rotation. Multiv Behav Res.

[30] Samejima F (1969). Estimation of Latent Ability Using a Response Pattern of Graded
Scores.

[31] Jordan P, Spiess M (2019). Rethinking the interpretation of item discrimination and factor
loadings. Educ Psychol Measur.

[32] Baker FB (2001). The basics of item response theory.

[33] Cronbach LJ (1951). Coefficient alpha and the internal structure of
tests. Psychometrika.

[34] McDonald RP (1999). Test theory: A unified treatment.

[35] Hoekstra R, Vugteveen J, Warrens MJ, Kruyen PM (2019). An empirical analysis of alleged misunderstandings of coefficient
alpha. Int J Soc Res Methodol.

[36] McNeish D (2018). Thanks coefficient alpha, we'll take it from here. Psychol Methods.

[37] Hancock GR, Mueller RO, Cudek R, duToit SHC, Sorbom DF (2000). Rethinking construct reliability within latent variable
systems. Structural equation modeling: Present and future.

[38] Ferrando PJ, Navarro-Gaonzáles D, Lorenzo-Seva U (2019). Assessing the quality and effectiveness of the factor score
estimates in psychometric factor-analytic applications. Methodology (Gott).

[39] Fokkema M, Greiff S (2017). How Performing PCA and CFA on the Same Data Equals
Trouble. Eur J Psychol Assess.

[40] Lee LC, Liong C-Y, Jemain AA (2018). Validity of the best practice in splitting data for hold-out
validation strategy as performed on the ink strokes in the context of
forensic science. Microchem J.

[41] Gütlein M, Helma C, Karwath A, Kramer S (2013). A Large-Scale Empirical Evaluation of Cross-Validation and
External Test Set Validation in (Q)SAR. Mol Inform.

[42] Lorenzo-Seva U, Van Ginkel JR (2016). Multiple imputation of missing values in exploratory factor
analysis of multidimensional scales: estimating latent trait
scores. Ann Psychol.

[43] Bollen KA (2019). When Good Loadings Go Bad: Robustness in Factor
Analysis. Struct Equ Model.

[44] Rodriguez A, Reise SP, Haviland MG (2016). Applying Bifactor Statistical Indices in the Evaluation of
Psychological Measures. J Pers Assess.

[45] Ingram RR, Kautz DD (2018). Creating "Win-Win" Outcomes for Patients with Low Health
Literacy: A Nursing Case Study. Med Surg Nursing.

